# Functional differences in human aortic valve interstitial cells from patients with varying calcific aortic valve disease

**DOI:** 10.3389/fphys.2023.1168691

**Published:** 2023-06-19

**Authors:** Robin Tuscher, Alex Khang, Toni M. West, Chiara Camillo, Giovanni Ferrari, Michael S. Sacks

**Affiliations:** ^1^ Department of Biomedical Engineering, James T. Willerson Center for Cardiovascular Modeling and Simulation, The Oden Institute for Computational Engineering and Sciences, The University of Texas at Austin, Austin, TX, United States; ^2^ Department of Surgery, The Seymour Cohn Cardiovascular Research Laboratory, Columbia University, New York, NY, United States

**Keywords:** aortic valve, bicuspid aortic valve, aortic valve interstitial cells, contractility, stress fibers, calcific aortic valve disease (CAVD), basal tonus

## Abstract

Calcific aortic valve disease (CAVD) is characterized by progressive stiffening of aortic valve (AV) tissues, inducing stenosis and insufficiency. Bicuspid aortic valve (BAV) is a common congenital defect in which the AV has two leaflets rather than three, with BAV patients developing CAVD decades years earlier than in the general population. Current treatment for CAVD remains surgical replacement with its continued durability problems, as there are no pharmaceutical therapies or other alternative treatments available. Before such therapeutic approaches can be developed, a deeper understanding of CAVD disease mechanisms is clearly required. It is known that AV interstitial cells (AVICs) maintain the AV extracellular matrix and are typically quiescent in the normal state, transitioning into an activated, myofibroblast-like state during periods of growth or disease. One proposed mechanism of CAVD is the subsequent transition of AVICs into an osteoblast-like phenotype. A sensitive indicator of AVIC phenotypic state is enhanced basal contractility (tonus), so that AVICs from diseased AV will exhibit a higher basal tonus level. The goals of the present study were thus to assess the hypothesis that different human CAVD states lead to different biophysical AVIC states. To accomplish this, we characterized AVIC basal tonus behaviors from diseased human AV tissues embedded in 3D hydrogels. Established methods were utilized to track AVIC-induced gel displacements and shape changes after the application of Cytochalasin D (an actin polymerization inhibitor) to depolymerize the AVIC stress fibers. Results indicated that human diseased AVICs from the non-calcified region of TAVs were significantly more activated than AVICs from the corresponding calcified region. In addition, AVICs from the raphe region of BAVs were more activated than from the non-raphe region. Interestingly, we observed significantly greater basal tonus levels in females compared to males. Furthermore, the overall AVIC shape changes after Cytochalasin suggested that AVICs from TAVs and BAVs develop different stress fiber architectures. These findings are the first evidence of sex-specific differences in basal tonus state in human AVICs in varying disease states. Future studies are underway to quantify stress fiber mechanical behaviors to further elucidate CAVD disease mechanisms.

## 1 Introduction

**FIGURE 1:**
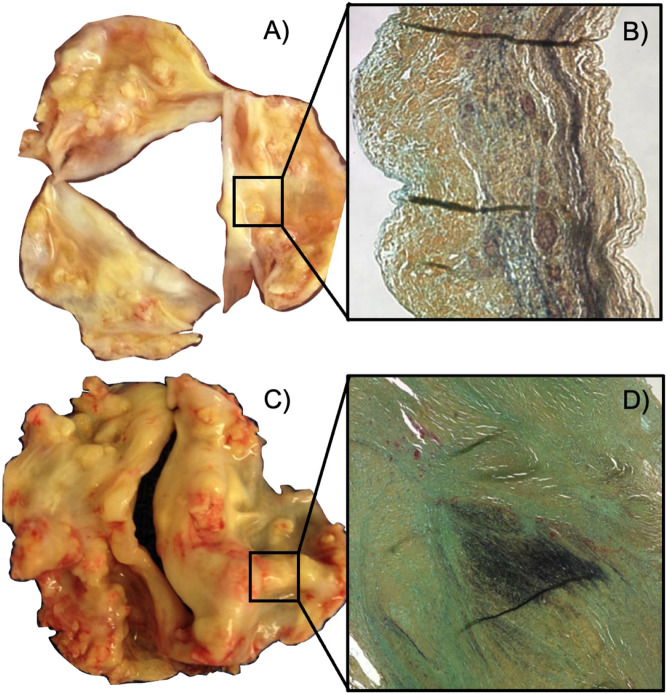
Changes in molecular structure between calcified tricuspid and bicuspid aortic valves. **(A)** Calcified tricuspid aortic valve. **(B)** Representative cross-sectional view of a tricuspid aortic valve leaflet. The tricuspid aortic valve has a neat, striated distribution of elastin (black). **(C)** Calcified bicuspid aortic valve. **(D)** Cross-sectional view of the raphe region of a bicuspid aortic valve. The bicuspid heart valve has clumped, disorganized elastin (black) in the raphe region (Aggarwal et al., 2014).

Calcific aortic valve disease (CAVD) is a progressive disorder of the aortic valve (AV) in which the aortic valve develops calcium deposits that gradually stiffen the AV and obstruct blood flow (Lerman et al., 2015). CAVD shares risk factors with artherosclerosis such as increased low-density lipoprotein (LDL) cholesterol, diabetes mellitus, smoking, and hypertension; however, CAVD has additional risk factors, one of which is a congenital valve defect known as bicuspid aortic valve (BAV) (Lerman et al., 2015; Braverman et al., 2005) (Figure 1C). BAV is present in roughly 1%–2% of the population with a 2:1 male prevalence and is characterized by the development of an aortic valve with two leaflets rather than the physiological three (Braverman et al., 2005) (Figure 1A). BAV defects are present in roughly 50% of patients undergoing AV replacement for aortic stenosis, implying a strong correlation between BAV and CAVD (Braverman et al., 2005; Lerman et al., 2015). In addition, patients with BAVs experience CAVD much earlier in life than patients with TAVs (Braverman et al., 2005). Braverman et al. (2005) reports that, of 932 patients undergoing AVR for aortic stenosis, two-thirds under 50 years old had a BAV and the remaining third had a unicuspid AV (Braverman et al., 2005). Two-thirds of patients between 50 and 70 years had a BAV, while only 40% had a BAV in patients over 70 years of age (Braverman et al., 2005).

For CAVD resulting in severe aortic stenosis, patients must receive an aortic valve replacement (AVR) (Lerman et al., 2015). Currently, there are no pharmaceutical treatments for CAVD (Lerman et al., 2015). There are two options for AVR in CAVD: bioprosthetic AVs and mechanical AVs (Head et al., 2017). These AVR types are prone to issues such as structural valve deterioration and high thrombogenicity, leading to reoperation and mortality (Jamieson et al., 1998; Bourguignon et al., 2015; Head et al., 2017; Tasoudis et al., 2022). Both AVR options have contraindications for a wide number of patients, demonstrating the need for a pharmaceutical treatment to delay or prevent progression of CAVD.

The leaflets of both TAV and BAVs are organized into three layers consisting of collagen, elastin, and glycosaminoglycans (Lerman et al., 2015). BAVs contain a region of highly disorganized microenvironment known as the raphe region, which lies along the center of the larger leaflet (Aggarwal et al., 2014; Rego et al., 2022) (Figure 1D). The raphe region contains the same relative composition of proteins as a TAV leaflet; however, these proteins are less aligned and much thicker than the TAV leaflet (Aggarwal et al., 2014). The corresponding mid belly region of a TAV has organized layers of aligned collagen (Aggarwal et al., 2014) (Figure 1B). Each layer of AV leaflet is interspersed with AV interstitial cells (AVICs) which have mechanosensory function and maintain the extracellular matrix of the leaflets. AVICs form *α*-smooth muscle actin (*α*-SMA) stress fibers that are responsible for their contractile function; AVICs attach to the surrounding microenvironment via integrins and sense the mechanical properties of the surrounding environment. In CAVD, the AVICs are induced into an osteoblast-like state and deposit calcium. Before this process occurs, the AVICs transition into an activated state in which contractility of the AVICs increases. For reasons not yet known, AVICs from BAVs are more likely to cause CAVD and likelier to cause early CAVD compared to AVICs from TAVs (Aggarwal et al., 2014). Aggarwal et al. (2014) has shown that the raphe region experiences different strains than TAV mid belly region which could cause AVIC activation (Aggarwal et al., 2014). In addition, Ayoub et al. (2021) has shown that exposing VICs to different peak stresses leads to changes in VIC shape and function.

Prior studies have investigated the intrinsic differences between VICs originating from the mitral, pulmonary, tricuspid, and aortic heart valves (Merryman et al., 2006; Merryman et al., 2007). Merryman et al. (2006) found that the left side heart VICs, i.e., mitral and aortic VICs, are stiffer than the right side heart VICs, i.e., tricuspid and pulmonary VICs, using micropipette aspiration (Merryman et al., 2006). In addition, AVICs were found to be stiffer than pulmonary VICs using atomic force microscopy (Merryman et al., 2007). These result suggests that exposure to higher transmural pressures causes VICs to become stiffer (Merryman et al., 2006; Merryman et al., 2007). Further, the connection between tissue-level loading and VIC response was elucidated by Lee et al. (2015), which demonstrated that applying different tensions to mitral VICs caused a change in nuclear aspect ratio and a preferred stress fiber direction (Lee et al., 2015). Studies have also studied the activation levels of AVICs in 3D. Human AVICs from non-calcified, tricuspid AV were shown to substantially increase *α*-SMA production when exposed to transforming growth factor *β* (TGF-*β*) (Duan et al., 2016). In addition to a change in expression, a change in contractility was found to increase in end-loading, flexural deformation testing of AVICs seeded in poly (ethylene glycol) hydrogel after addition of hypertensive KCl Khang et al. (2019). Functional differences between bicuspid and tricuspid AVs in full 3D have not been studied. Likewise, differences between human diseased AVICs from different AV regions have not been studied.

Stress fibers are a dynamic structure consisting of actin-myosin cross bridges, actin filaments, and integrin-mediated anchoring to the cytosol and hydrogel. Actin monomers are continuously added and removed from the stress fiber (Sakamoto et al., 2017). In a highly contractile state, *α*-SMA is selectively added to the stress fiber (Sakamoto et al., 2017), increasing the contractility of the AVIC. Both the force per unit length strength fiber and the mass fraction of stress fiber in a particular region of AVIC can determine basal tonus, or baseline level of contraction exhibited by AVICs. Stress fibers can be imaged in 2D, but no modality exists that can resolve stress fibers in full 3D such that functional differences may be observed. Basal tonus is thus a functional metric which correlates with the level of activation in AVICs.

This study is the inital step to addressing the paucity of knowledge of different AV types and regions. AVICs were isolated from patients with CAVD with both BAV and TAV morphology. AVICs from the raphe region of BAVs (RBAVs), AVICs from the BAV non-raphe region (NRBAV), AVICs from a calcified region of a TAV (CTAV), and AVICs from a non-calcified region of a TAV (NCTAV) were studied. Isolating AVICs from different regions of the same valve may elucidate whether the AVICs themselves or the strain state of the origin cause differences. The purpose of this study was to determine whether the AVICs from diseased patients are intrinsically more activated or activated they originate from a diseased microenvironment. Rego et al. (2022) has shown that stretches differ significantly between different regions of the same leaflet of AV valves (Rego et al., 2022). These effects have been shown to cause different deformations in affected AVICs, which may cause differing activation levels (Ayoub et al., 2017). By culturing the AVICs in identical PEG hydrogels, the intrinsic activation in the same microenvironment may be studied. This study identifies some crucial differences in the stress fiber architecture between AVICs from different regions of calcified AVs.

## 2 Methods

### 2.1 AVIC sourcing, extraction, and culture

#### 2.1.1 Human diseased AVICs from TAVs and BAVs

The Institutional Biosafety Committee of the University of Texas, Austin approved all protocols for experimentation (IBC-2021-00047) in accordance with NIH guidelines. De-identified human AVIC samples were obtained from Columbia University Biobank for Translational Studies under IRB protocol *#*AAAR6796, which were isolated as previously described (Stephens et al., 2007). AVICs from the raphe region of BAVs (RBAVs), AVICs from the BAV non-raphe region (NRBAV), AVICs from a calcified region of a TAV (CTAV), and AVICs from a non-calcified region of a TAV (NCTAV) were studied. A detailed account of the number of AVIC samples is shown in Table 1. All materials and reagents were procured from Thermo-Fisher Scientific unless otherwise specified.

**TABLE 1:** Cell bank of diseased human AVICs from TAV and BAV patients with CAVD for each experimental group. The number of AVICs in each category is shown separated into AV physiology, location of leaflet, and sex.

Tricuspid				Bicuspid			
Non-Calcified		Calcified		Non-Raphe		Raphe	
Male	Female	Male	Female	Male	Female	Male	Female
6	6	8	5	12	6	11	6

#### 2.1.2 Cell sourcing and processes

Passage 0 (p0) human AVICs from specific patients were cultured from 2-mL frozen aliquots of freeze media into rat tail collagen-coated T25 flasks in DMEM + 10% FBS + 1x PSG (Gibco) until cells were 85% confluent. Confluent p0 cultures were passaged into rat tail collagen-coated T75 flasks for cell expansion and frozen in freeze media (Sigma). p1 cells were grown to confluence in collagen-coated T25 flasks. The cell membranes were stained with CellBrite Red (Biotum, Hayward, CA) immediately before AVICs were trypsinized for p2 implementation during experimentation.

#### 2.1.3 3D PEG hydrogels for culture and microscopy

As previously reported in Khang et al. (2022a), AVICs were embedded in a poly (ethylene) glycol (PEG) gel for conducting 3D-TFM experimentation. To produce the PEG gel, a pre-polymer solution containing 333,000 cells/mL, 3% 8-arm 40 kDa PEG-norbornene (Jenkem), 1 mM CRGDS adhesive peptides (Bachem), 0.05% lithium phenyl- 2,4,6—trimethylbenzolphosphinate (LAP) photoinitiator (Millipore-Sigma), and 1.672 mM MMP-degradable crosslinker (Bachem) in dPBS was placed in the bottom of the glass portion of a 35 mm imaging dish. In addition, for imaging gel displacements, 0.5 *μ*m yellow-green fluorescent microspheres (Polysciences, Warrington, PA) were suspended at a density of 3 × 10^9^ spheres/mL (Khang et al., 2022a). The gel was cross-linked for 3 min with UV light (365 nm, 2.5 mW/cm^2^). The resultant gels containing live cells were cultured for 3 days in DMEM + 10% FBS + 1x PSG (Gibco) so that the AVICs could integrate into the gel before experimentation. The modulus of the gel was ≈ 100 Pa (Khang et al., 2023).

### 2.2 Experimental measurements

#### 2.2.1 Three-dimensional traction force microscopy (3D-TFM)

AVICs were imaged with a Zeiss 710 Laser Scanning Microscope equipped with a 63x water immersion lens while implementing 488 and 640 lasers in succession to separately capture the cell membrane stain and fluorescent markers at the Center for Biomedical Research Support Microscopy and Imaging Facility at UT Austin (RRID*#* SCR_021756) (Khang et al., 2022a). Within a temperature-controlled chamber on the microscope, samples were equilibrated for 40 min in Tyrode’s solution, imaged, treated with 4 *μ*M cytochalasin-D (CytoD) in Tyrode’s for 40 min, and then re-imaged.

### 2.3 Data analysis

#### 2.3.1 TFM marker recovery and marker displacement

FM-Track software was used to recover marker positions and displacements (Khang et al., 2019; Lejeune et al., 2020). FM-Track uses a nearest neighbor approach to track beads from one image set to the next, producing a one-to-one point correspondence between the normal state (N), i.e., no treatment, and inactivated state (I), i.e., CytoD treatment. FM-Track first locates the fluorescent markers at each slice of the z-stack in the N and I states. Next, FM-Track identifies the same markers from the N and I states by associating the markers with its five to fifteen nearest neighbors. The unique combination of neighbors is sufficient to identify the same bead in both states. Then, any marker displacement from one state to the next is calculated and recorded. The AVIC surface boundary is located using marker displacements cell membrane staining. The membrane images are isolated and confirmed against the positions of markers. In addition, surface displacements are confirmed with the marker displacements.

#### 2.3.2 AVIC surface mesh recovery

AVIC surface meshes were produced from the membrane images using several post-processing steps and checks. First, the 488 channel raw. tif images were compiled into vertices and connectivity matrices of size 3 ×n, where n is equivalent to the number of points in 3-space that produce the mesh. The main cell geometry is determined by selecting the largest amalgam of connected volumes. In the case of exceedingly long protrusions that are truncated by the binary closing, the pre-closed geometry is re-added to the closed geometry. The final AVIC mesh is then smoothed via MeshLab processing. This procedure was repeated for all AVIC images in both N and I states.

#### 2.3.3 Analysis of AVIC shape

Based on previous work (Khang et al., 2022a), spherical harmonics (SH) were used to quantitatively assess the AVIC surfaces. The SPHARM-PDM (https://www.nitrc.org/projects/spharm-pdm) extension of SlicerSALT software (https://www.slicer.org/) was used to fit a SH model to the AVIC surface meshes. SPHARM-PDM uses an ellipsoid of best fit to align the AVIC surface meshes with their principal directions, represented as *x*_1_, *x*_2_, and *x*_3_. Once aligned, SPHARM-PDM models each AVIC separately in the N state and the I state. The model returns fitted SH series with SH coefficients $\left(C_{l}^{m}\right)$ unique to a specific degree *l* and order *m*. ClximN is defined as the SH coefficient for degree *l* and order *m* in an N state AVIC in the *x*_*i*_ direction. ClximI is defined as the SH coefficient for degree *l* and order *m* in an I state AVIC in the *x*_*i*_ direction. The AVIC surface meshes were reconstructed with the fitted coefficients to a given*l* and *m*.

#### 2.3.4 Computation of AVIC shape change

The change in overall AVIC shape has been shown to correlate with the change in contractility of the AVIC (Khang et al., 2022a). Moreover, the total shape change is a combined metric of AVIC basal contractile state with the compensatory isovolumetric changes in response Cytochalasin-D. However, AVICs form complicated protrusions in 3D culture, requiring a method that determines shape change independent of starting shape. SH series were used to compute this change in shape in a way that is robust to variations in initial shape (Khang et al., 2022a). The change in shape was calculated as the difference between SH coefficients for each order and degree (Eq. 1). The total difference for any direction was computed as $\Delta_{x_{i}}$ as shown in Eq. 2. The overall shape change of the cell for all directions was computed as $\bar{\Delta }$ as shown in Eq. 3. The change in SH coefficients in the second and third principal directions relative to the first principal direction was calculated using Eq. 4 and Eq. 5.

Δlxim≡ClximN−ClximIfori=1,2,3 (1)

$$\Delta_{x_{i}}\equiv \overset{10}{\underset{l=1}{\sum }}\overset{l}{\underset{m=-l}{\sum }}\Delta_{l_{x_{i}}}^{m}$$ (2)

$$\begin{array}{ll} \bar{\Delta } & =\underset{\text{all cells}}{\text{mean}}\left(\sqrt{\overset{3}{\underset{i=1}{\sum }}{\left(\overset{10}{\underset{l=1}{\sum }}\overset{l}{\underset{m=-l}{\sum }}\Delta_{l_{x_{i}}}^{m}\right)}^{2}}\right)\\ & =\underset{\text{all cells}}{\text{mean}}\left(\sqrt{{\left(\Delta_{x_{1}}\right)}^{2}+{\left(\Delta_{x_{2}}\right)}^{2}+{\left(\Delta_{x_{3}}\right)}^{2}}\right) \end{array}$$ (3)

$$\frac{\Delta_{x_{2}}}{\Delta_{x_{1}}}=\underset{\text{all cells}}{\text{mean}}\left(\frac{\overset{10}{\underset{l=1}{\sum }}\overset{l}{\underset{m=-l}{\sum }}\Delta_{l_{x_{2}}}^{m}}{\overset{10}{\underset{l=1}{\sum }}\overset{l}{\underset{m=-l}{\sum }}\Delta_{l_{x_{1}}}^{m}}\right)$$ (4)

$$\frac{\Delta_{x_{3}}}{\Delta_{x_{1}}}=\underset{\text{all cells}}{\text{mean}}\left(\frac{\overset{10}{\underset{l=1}{\sum }}\overset{l}{\underset{m=-l}{\sum }}\Delta_{l_{x_{3}}}^{m}}{\overset{10}{\underset{l=1}{\sum }}\overset{l}{\underset{m=-l}{\sum }}\Delta_{l_{x_{1}}}^{m}}\right)$$ (5)

The 3D nuclear aspect ratio was computed as shown in Eq. 6. This metric represents shape change in three principal directions.

$$NAR=\frac{{\left(\Delta_{x_{2}}\right)}^{2}}{\Delta_{x_{1}}\Delta_{x_{3}}}$$ (6)

#### 2.3.5 Surface displacements

Prior studies have found that the nature of deformation at the tips of the protrusions is piston-like (Khang et al., 2022a). Thus, the surface displacements primarily occur outwards in a direction normal to the cell surface at the tip of the protrusion. These displacements were computed first by isolating the tip of the protrusion. Protrusion tips were used because they displace primarily normal to the AVIC surface, thus measuring the change in stress fiber length the most accurately. Surface displacement vectors $\left(\underline{u}\right)$ were computed using Eq. 7 from FM-Track’s surface displacements.

$$\underline{u}\equiv u_{1}\hat{i}+u_{2}\;\hat{j}+u_{3}\hat{k}$$ (7)

The average *ℓ*_2_ norm for each protrusion was calculated for the surface displacement vectors using Eq. 8.

$$\bar{u}=\underset{\text{all cells}}{\text{mean}}\left(\|\underline{u}{\|}_{2}\right)=\underset{\text{all cells}}{\text{mean}}\left(\sqrt{{\left(u_{1}\right)}^{2}+{\left(u_{2}\right)}^{2}+{\left(u_{3}\right)}^{2}}\right)$$ (8)

For each AVIC, the largest surface displacements were isolated. The volume enclosed by the isolated points was checked so that it was not larger than 0.5% of total AVIC volume. A principal component analysis (PCA) was run on the points, followed by a K-means clustering. The number of clusters was determined by visual inspection. Each cluster was lastly inspected and selected if it matched to the tip of an AVIC protrusion (Supplementary Figure S11). The means for each protrusion tip were computed, and between one and three protrusions were analyzed for each AVIC. The surface clips were compared against manual protrusion clips using Paraview software.

#### 2.3.6 In-plane AVIC membrane deformations

Stretches were computed from AVIC membrane displacement fields computed in FM-Track as done in prior studies (Khang et al., 2022a). In brief, we relied on the ability for FM-Track to track cell surface deformations using a finite element mesh where each mesh node was represented as a convected material point. This was done by interpolating the local gel field displacements onto the nodes of the AVIC surface mesh using Gaussian Process Regression (Lejeune et al., 2020). We then developed a local in-surface coordinate system which allowed us to compute in-surface metric tensors in the N to I states. From the metric tensors the in-surface principal stretches ( ${\left(\lambda_{\text{I}}\right)}^{2}$ and ${\left(\lambda_{\text{II}}\right)}^{2}$ ) were computed. The resulting principal stretches were then assessed for magnitude differences between experimental groups and sex.

## 3 Results

### 3.1 AVIC shape change with relaxation

#### 3.1.1 Trends in shape change

**FIGURE 2:**
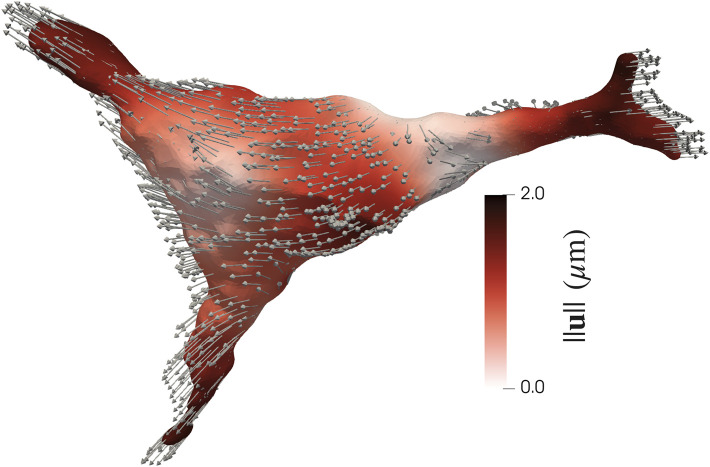
Shape change induced by inactivation by CytoD. **(A)** Normal (gray) to Inactivated (green) shape change for a representative AVIC. **(B)** Change in SH coefficient from Normal to Inactivated states is mostly negative (Eq. 1). **(C)** Inactivated AVIC reconstructed to 50° of spherical harmonics. **(D)** Inactivated AVIC reconstructed to 10° of spherical harmonics, capturing the same shape of the AVIC reconstruction using 50° of spherical harmonics.

**FIGURE 3:**
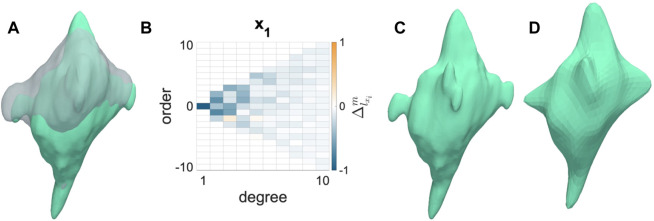
Change in spherical harmonic coefficient ( $\Delta_{l_{x_{i}}}^{m}$ , Eq. 1) for each diseased AVIC group. Separate plots for each principal direction are shown. The maximal change is exhibited in the *x*_1_ plot. All compensatory change to the positive direction is exhibited in the *x*_3_ plot. **(A)** NCTAV group exhibits the largest magnitude of change. **(B)** CTAV group exhibits the next highest magnitude of change. **(C)** RBAV group exhibits the next highest magnitude of change. **(D)** NRBAV group exhibits the lowest magnitude of change.

AVICs from all groups became less spherical and more dendritic from the N to I states (Figure 2A). AVIC surface meshes were reconstructed to 50° and 10° of SH terms. SH coefficients for a particular degree (*l*) and order (*m*) correspond with the contribution of a particular orbital with degree *l* and order *m* to the AVIC surface mesh reconstruction. Increasing degree and order increase the complexity of the orbital; thus, the magnitude of higher order terms for the I AVIC reconstruction was greater than the magnitude of those terms for the N AVIC reconstruction. For both N and I, the difference in SH coefficients beyond (10°) approached zero and became negligible (Figures 2B, 3). Higher order terms in the SH series represent surface roughness, which stayed relatively the same for both the N and I states (Figures 2C, D). AVIC surface reconstructions without the higher order terms still capture the basic shape of the AVIC (Figures 2C, D). Further computations of total AVIC shape change were performed with SH coefficients between to 0° and 10°.

#### 3.1.2 Magnitude of shape change

**FIGURE 4:**
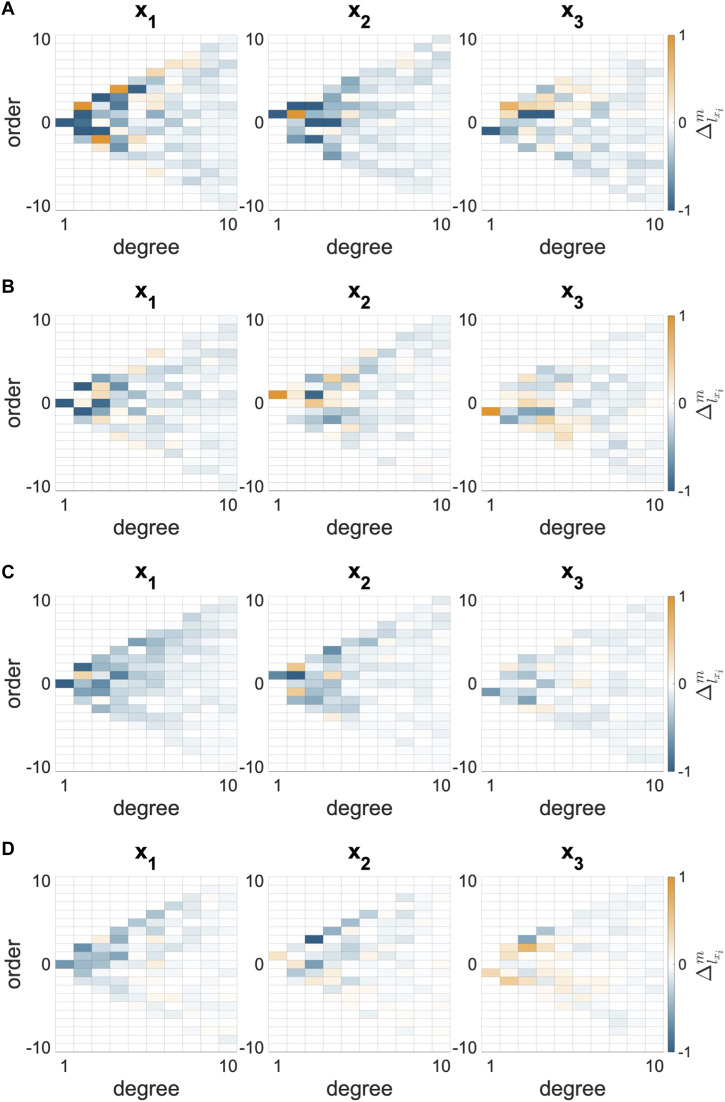
Average change in SH coefficient from N to I states for the whole AVIC 3. The NCTAV group has the largest average magnitude of total shape change. The CTAV and RBAV groups were statistically similar. The NRBAV group had the smallest average magnitude of total shape change. P-value*<0.05,P-value**<0.005 .

The differences in SH coefficients ( $\Delta_{l_{x_{1}}}^{m}$ , Eq. 1) were negative for all groups in the principal (*x*_1_) direction of relaxation (Figure 3). This difference reflects that the N AVIC surface meshes are more spherically shaped than the I AVIC surface meshes, which have longer protrusions. The total magnitude of shape change, $\bar{\Delta }$ , was calculated as a root-mean-squared value for all directions (Eq. 2). This metric roughly represents the whole-cell change in AVIC shape from N to I states. The NCTAV group had the largest average magnitude of total shape change ( $\bar{\Delta }$ , Eq. 3; Figure 4). The CTAV and RBAV groups had the next largest average magnitude of total shape change. The NRBAV group had the least average magnitude of total shape change.

#### 3.1.3 Polarity of shape change

**FIGURE 5:**
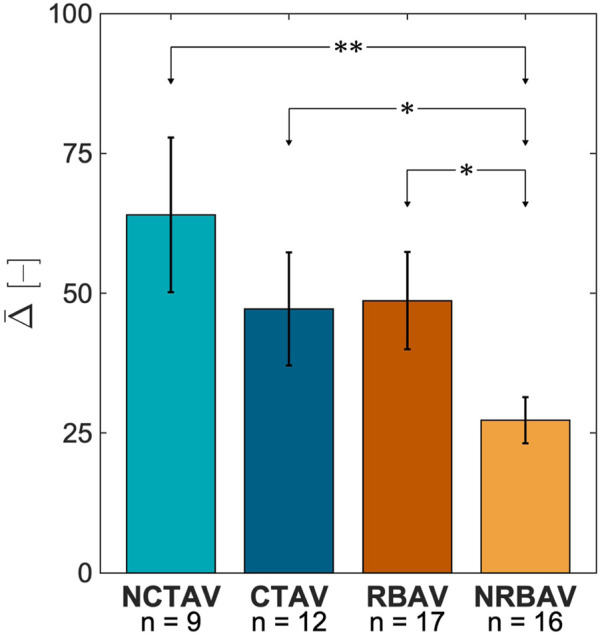
Total AVIC shape change in spherical harmonic coefficient normalized to the principal axis. **(A)** Ratios of shape change ( $\frac{\Delta_{x_{2}}}{\Delta_{x_{1}}}$ , Eq. 4; $\frac{\Delta_{x_{3}}}{\Delta_{x_{1}}}$ ; Eq. 5). **(B)** Representative ellipsoid deformations. The TAVICs deformed equally in the *x*_1_ and *x*_2_ principal directions, while the BAVICs deformed primarily in the *x*_1_ principal direction. P-value*<0.05,P-value**<0.005 .

The directional shape change was also computed using the SH analysis. The greatest magnitude of shape change in all groups occurred in the *x*_1_ direction which is aligned with the longest axis of the AVIC (Figure 5A). To compare the relative change in shape in the three principal directions, the ratio of shape change in the second and third principal directions relative to the first principal direction was calculated (Eq. 4 and Eq. 5). For a ratio near one, the deformation in that direction is roughly equal to the deformation in the first principal direction. A ratio less than one indicates less deformation in that direction than in the first principal direction. The NCTAV group experienced roughly equal deformations in the first and second principal directions and approximately half as much deformation in the third principal direction (Figure 5A; Table 2). Likewise, the CTAV group experienced roughly 40% of the first and second principal deformations in the third principal direction (Figure 5A; Table 2). The RBAV group experienced the maximum deformation in the first principal direction, and roughly equal deformation in the second and third principal directions (Figure 5A; Table 2). Both the second and third principal directions relaxed 50% as much as the first principal direction (Figure 5A; Table 2). Similarly, the NRBAV group experienced roughly equal deformation in all directions and the most in the first principal direction (Figure 5A; Table 2). AVICs in the tricuspid groups (NCTAV and CTAV) relaxed in a oblate transverse isotropic manner (Figure 5B). AVICs in the bicuspid groups (RBAV and NRBAV) relaxed in a prolate transverse isotropic manner (Figure 5B).

**TABLE 2:** 95% confidence interval of proportion of SH shape change (Eq. 4; Eq. 5) in the second and third principal directions relative to the first principal direction. The TAVICs exhibit oblate transverse isotropic behavior because most SH shape change occurs in the first two principal directions. The BAVICs exhibit prolate transverse isotropic behavior since most shape change occurs in the first principal direction.

	NCTAV	CTAV	RBAV	NRBAV
$\Delta_{X_{2}}:\Delta_{X_{1}}$	1.07 ± 0.40	0.85 ± 0.65	0.56 ± 0.37	0.88 ± 0.59
$\Delta_{X_{3}}:\Delta_{X_{1}}$	0.51 ± 0.41	0.36 ± 0.34	0.50 ± 0.36	0.67 ± 0.48

### 3.2 Surface protrusion displacements

**FIGURE 6:**
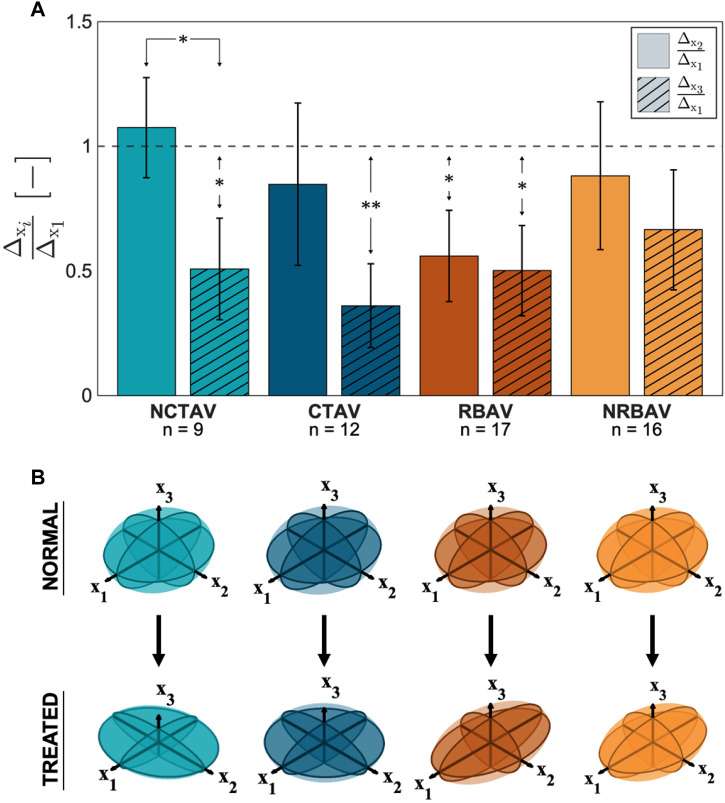
Magnitude of surface displacements of an AVIC (Eq. 7). The largest displacements occur at the tips of the protrusions in a direction outward the center of the AVIC, as indicated by the displacement vectors.

The tips of the protrusions have the largest magnitude of displacement (Figure 6). Thus, the displacements at the tips of the protrusions were computed and compared amongst experimental groups (Supplementary Figure S11).

#### 3.2.1 Magnitude of displacement

**FIGURE 7:**
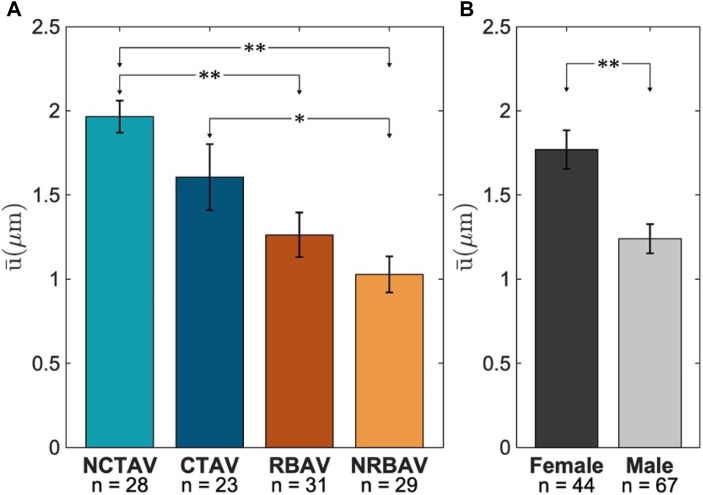
Average displacement at tips of the protrusions. **(A)** Whole view of AVIC. **(B)** Sex-specific differences between females and males. P-value*<0.05,P-value**<0.005 .

The NCTAV group had the largest average displacement (Figure 7A). This result concurs with the finding that the NCTAV group has the largest magnitude of overall shape change. The next largest displacement is the CTAV group, followed by RBAV. The least magnitude of displacement is the NRBAV group.

#### 3.2.2 Sex-specific differences

The surface displacements at the tip of AVIC protrusions were found to be on average 0.5 *μ*m larger in females than in males (Figure 7B).

### 3.3 AVIC membrane stretches at the protrusions

**FIGURE 8:**
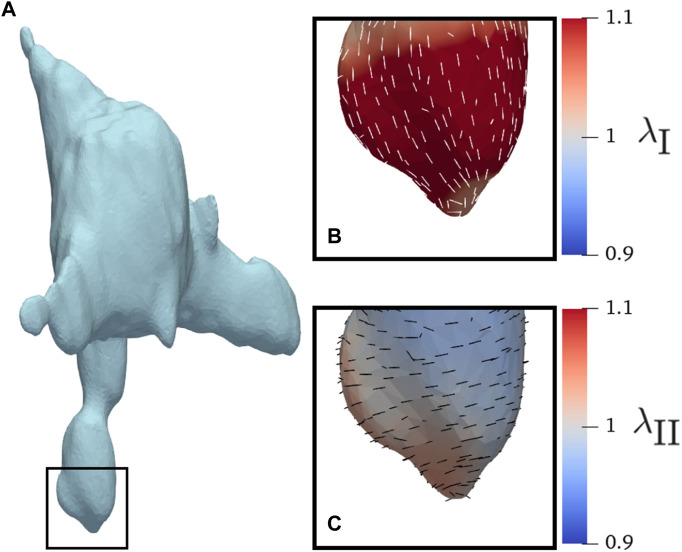
AVIC membrane stretches of a human AVIC. **(A)** Whole view of AVIC.**(B)** Principal stretch *λ*_I_, and **(C)** Minor stretch *λ*_II_.

**FIGURE 9:**
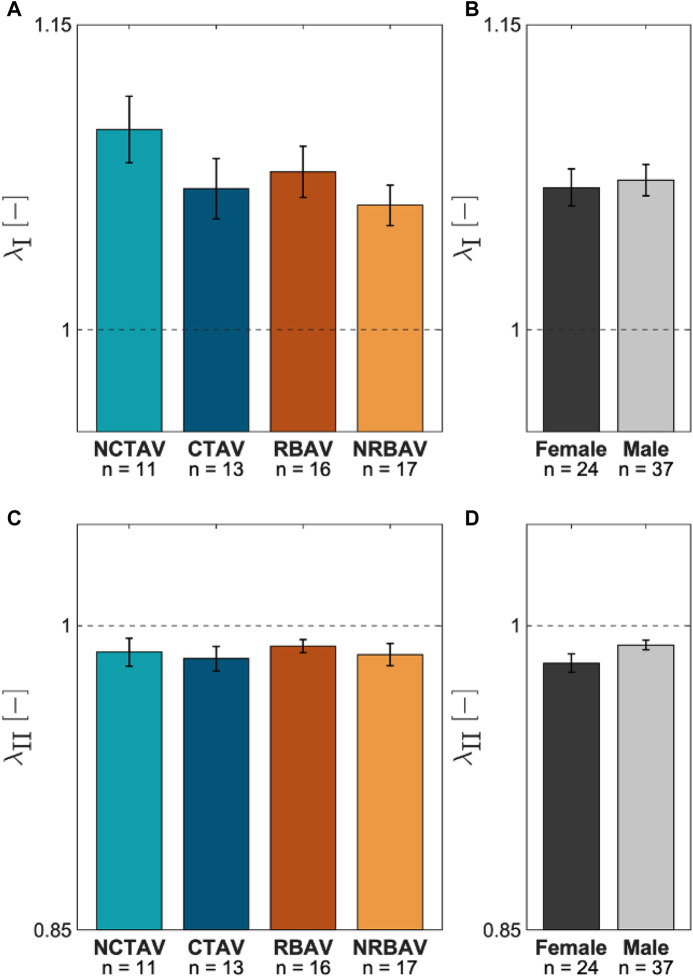
Average AVIC membrane stretch at the protrusions. **(A)** Comparison between groups for *λ*_I_, the first principal stretch. **(B)** *λ*_I_ comparison between sexes. **(C)** Average *λ*_II_, or second principal stretches, for the experimental groups. **(D)** *λ*_II_ comparison between sexes.

In-plane surface membrane stretches were computed for the protrusions (Figure 8A). The major stretches typically aligned along the longitudinal direction (Figure 8B). The minor stretches typically aligned in the circumferential direction (Figure 8C). There was no significant difference in magnitude of stretch between the AVIC groups (Figure 9). As expected, the AVICs lengthened in the longitudinal direction and shrunk in the circumferential direction.

### 3.4 Polarity and contraction distance

**FIGURE 10:**
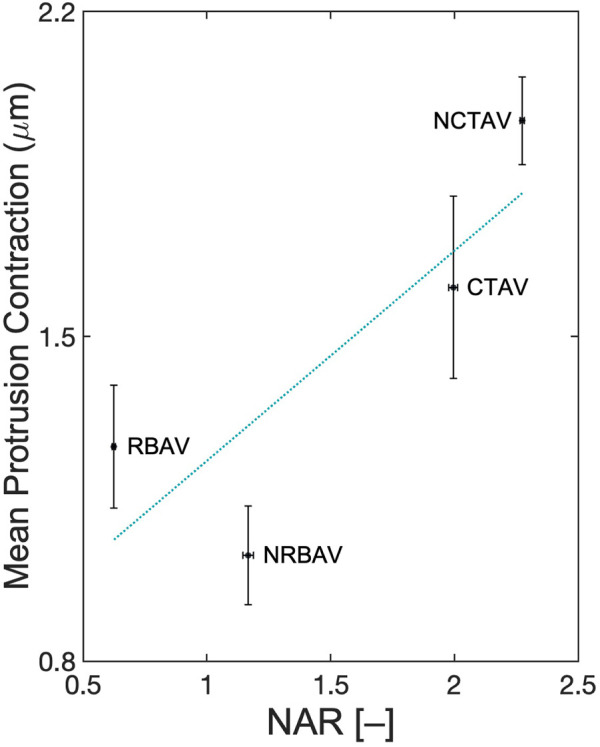
Correlation between polarity and average contraction along protrusions. 3D-NAR is defined as shown in Eq. 6. A linear least squares regression was performed. The line of best fit has equation x2 = 0.45 ⋅ x1 + 0.78 where x1 is the NAR and x2 is the mean protrusion contraction. R2 = 0.70.

Polarity was compared to contraction distance to determine if there was a relation between the two. Polarity was defined as the variation in shape change between principal directions computed as a 3D nuclear aspect ratio (NAR). Eq. 6 was used to compute the NAR. The NAR was lower in AVICs with a single polar direction and higher in AVICs with two polar directions. The NAR correlated positively with average contraction along protrusions with a squared correlation coefficient of 0.70 (Figure 10).

## 4 Discussion

Changes in overall shape of the AVIC from a normal state to an inactivated state correspond with the basal tonus, or intrinsic contractility of the AVIC. In addition to the total shape change, the crucial areas of shape change for AVICs were in the protrusions. Each protrusion occurs in an area of heightened stress fiber alignment and integrin attachment. Both the tips of the protrusion and the arm of the protrusion were studied and compared between groups.

### 4.1 Primary findings

Significant functional differences between AVICs originating from TAVs and BAVs were demonstrated. As all AVICs were evaluated in the same environment, the observed differences in function suggest *intrinsic* differences in AVICs derived from TAVs and BAVs. Novel to this study was the demonstration of sex-specific functional differences underlying these intrinsic differences in basal tonus in human AVICs. The results of this study provide evidence that CAVD progression might derive from both native AVIC phenotypic state and AVIC microenvironment.

### 4.2 Basal tonus differs between groups

The overall change in AVIC shape indicated the basal level of stress fiber contraction. This is because after application of Cytochalasin D, all resultant deformation is due to depolymerization of stress fibers. Lam et al. (2016) has shown previously that elongated actin filaments correlate with *α*-SMA production. Additionally, basal contraction force has been shown to increase with the upregulation of *α*-SMA and alignment of stress fibers (Lam et al., 2016). This study was able to expand these results with human AVICs in full 3D. The largest overall shape change occurred in the NCTAVICs, indicating a higher basal tonus than the other experimental groups. Since these AVICs are intrinsically activated, they form protrusions which tether to the surrounding hydrogel via integrins and contract the gel. After the addition of Cytochalasin D, the contractile stress fibers are inactivated and the protrusions subsequently relax outwards from the AVIC center of mass. The cell body is likely less tethered to the microenvironment than the protrusions since the AVIC membrane collapses inwards as the protrusions relax outwards to maintain a constant cell volume. Thus, both the deformation of the AVIC membrane and the relaxation at the protrusions are both components of overall AVIC shape change. NRBAV AVICs had the least deformation, indicating the lowest basal tonus of the experimental groups. A possible explanation is that the AVICs from a calcified region of TAVs are further progressed into osteoblast-like differentiation than from a non-calcified region. Thus, these CTAV AVICs are stiffer and less contractile than the NCTAV AVICs.

### 4.3 Stress fiber architecture differs between groups

TAVICs and BAVICs exhibited functional differences that reflect differences in stress fiber architecture and function. Overall, the greatest magnitude of AVIC shape change occurred in the same direction as the AVIC’s longest axis. This finding suggests that the longest protrusions experience the greatest change in shape, which supports findings that demonstrate protrusions contain most of the contractile machinery of the AVIC (Khang et al., 2019). Deformation along all three principal axes was analyzed to determine relative alignment of stress fibers. CytoD treatment has been shown in prior studies to reduce the alignment of stress fibers (Sakamoto et al., 2016; Sakamoto et al., 2017). Thus, any deformations in distinct directions from N to I indicates prior alignment in those directions. The proportion $\frac{\Delta_{x_{2}}}{\Delta_{x_{1}}}$ (Figure 5A) represented the change in the second principal direction relative to the first principal direction. Likewise, $\frac{\Delta_{x_{3}}}{\Delta_{x_{1}}}$ (Figure 5A) represents the change in the third principal direction relative to the first principal direction. Together, these metrics represent the net effect of alignment of stress fibers in each AVIC group. For the TAV AVICs, around 50% of the first principal deformation occurred in the third principal deformation, suggesting that fewer stress fibers are aligned in that third principal direction than in the first two. The TAV AVIC stress fibers may branch in an oblate transversely isotropic manner. For the RBAV AVICs, roughly 50% of the relaxation occurred in the second and third principal directions. Thus, the RBAV AVICs may prefer to align stress fibers along one principal direction, giving rise to a prolate transversely isotropic relaxation. Past studies have shown that anisotropy is elevated in highly activated VICs (Lam et al., 2016; West et al., 2023). Lam et al. (2016) studied VICs cultured in 2D coverslip molds of varying aspect ratio. The results shown here expand upon this result by using 3D culture and capturing intrinsic anisotropy in diseased AVICs. The results agree with the finding that anisotropy is expected in activated AVICs, and add a third and final direction for comparison. Ultimately, all four AVIC groups displayed anisotropy between their first and third principal axes.

The stress fiber networks differed in alignment and contractility. There was a positive correlation found between the 3D-NAR and the contractility of the AVICs (Figure 10). A positive correlation suggests that the number of directions along which stress fiber networks arrange coincides with an increase in contractility. Highly polar cells like RBAVICs, which have a single direction of basal contraction, exhibited less contraction than cells with two principal directions of basal contraction like NCTAVICs.

### 4.4 Sex-specific differences in AVIC activation

Female AVICs were found to be more activated on average than male AVICs. This result agrees with prior research suggesting that female VICs express more *α*-SMA than male VICs (Aguado et al., 2022). *α*-SMA recruitment into stress fibers is a metric of AVIC activation and can increase contraction force and focal adhesions (Hinz et al., 2003; Sakamoto et al., 2016). The techniques used in this study indicate a difference in contraction strength rather than number of focal adhesions, as the amount of expressed integrins on AVIC surface is hypothesized to be greater than the number of CRGDS binding sites in the gel; thus, each AVIC is thought to bind the gel to saturation (Khang et al., 2019). Although Aguado et al. (2022) shows that female pAVICs are more sensitive to hypercontraction via endothelin-1 (ET-1) and the human AVICs in this study were not activated with ET-1, all AVICs used are activated natively by virtue of originating from diseased, calcified AVs (Aguado et al., 2022).

### 4.5 AVIC membrane stretches suggest mode of inactivation

The AVIC membrane stretches indicate there is no significant difference between the experimental groups. Our findings agree with previous publications that show piston-like deformation at the protrusions (Khang et al., 2019). The stretches are assessed along the protrusions, where the maximal relaxation occurs. The major stretches aligned along the longitudinal direction, indicating a relaxation along the length of the protrusion. The minor stretches aligned along the circumferential direction, indicating narrowing of the protrusion as relaxation occurs. The narrowing likely occurs isovolumetrically, preserving the volume of the AVIC despite lengthening of the protrusions. Although the displacement at the tips of the protrusions was greatest in the NCTAV group and least in the NRBAV group, the membrane stretches do not reflect the trend to the same extent. The NCTAV group has the largest longitudinal stretch, though not sufficiently large to fully explain the displacements at the tip of the protrusion. Thus, the relaxation likely occurs similarly to a piston: a contracted protrusion relaxes linearly outwards in a direction normal to the piston face.

### 4.6 Differences in AVIC groups due to lasting micro-environmental differences

As stated above, the extracellular environment was identical for all AVIC groups. As the amount of expressed integrins on AVIC surface is hypothesized to be greater than the number of CRGDS binding sites in the gel, each AVIC is thought to bind the gel to saturation (Khang et al., 2019). In addition, the stiffness of the gel is consistent between AVIC groups. Thus, the measured and computed differences between AVICs are attributed to lasting effects from the disease state of origin, not from differences in culture microenvironment.

### 4.7 Limitations

As with any investigation this study had several limitations. First, we note that while producing statistically significant findings, the numbers of human AVICs were limited. This was a result due to the exploratory nature of this study and the limited number of human AVICs available. We also note that we were not able to evaluate AVICs from human normals due to the lack of availability. We did attempt to provide a reference point through the use of healthy porcine AVICs from adult animals. When compared to the present data, the diseased human AVICs were much more activated than the healthy porcine AVICs, exhibiting 2–5 times more net shape change than the porcine AVICs (Supplementary Figure S16). This finding is consistent with the greater activation levels in diseased AV, but future work with human normals will be required.

### 4.8 Future directions

This study demonstrated the first evidence of differences between males and females in the contractility of human AVICs. The findings suggest there are functional differences between AVICs originating from varying disease states and phenotypes. Fundamentally, contractility in this study represents the ability of stress fibers in an activated AVIC to generate force. AVIC contraction is a complex interaction between cyto-skeletal filaments and motor proteins which are not possible to image in full resolution in 3D. Thus, study of these structures requires simulations to determined the stress fiber dispersion and contraction forces continuously throughout the AVIC.

In order to do this, we will first need to quantify how the AVICs remodel the gel, as the precise local gel behaviors are required for inverse modeling of the TFM experiment. However, the local stiffness of the hydrogel is difficult to measure directly. Ambiguity in hydrogel mechanics can lead to large errors in computed cellular tractions. We have recently developed an inverse computational approach to estimate AVIC-induced remodeling of the hydrogel materials (Khang et al., 2022b). When applied to AVICs assessed via 3DTFM, this approach estimated spatially separate regions of significant stiffening and degradation in the vicinity of the AVIC. We observed that stiffening was largely localized at AVIC protrusions, a result of collagen deposition as confirmed experimentally. Degradation was more spatially uniform and present in regions further away from the AVIC, likely a result of enzymatic activity during the 3 days incubation period. Using these novel results, simulations of the AVIC stress fiber structure revealed the largest forces and highly aligned fibers in the AVIC protrusions (Khang et al., 2022b), consistent with the observed large displacements in these regions. Ongoing studies are currently being conducted on the present dataset to reveal changes in stress fiber forces and structures between the groups.

This study also demonstrated the first evidence of differences between males and females in the contractility of human AVICs. In addition, the findings suggest there are functional differences between AVICs originating from varying disease states and phenotypes. Contractility in this study represents the ability of stress fibers in an activated AVIC to generate force. AVIC contraction is a complex interaction between cytoskeletal filaments and motor proteins which are not possible to image in full resolution in 3D. Thus, study of these structures requires biomechanical simulation to study in 3D. Future studies will investigate the dispersion of stress fibers and the contraction force continuously throughout the AVIC.

## Figures and Tables

**Table:** 

**AV**	Aortic valve
**CAVD**	Calcific Aortic Valve Disease
**TAV**	Tricupsid Aortic Valve
**BAV**	Bicuspid Aortic Valve
**NCT**	Non-Calcified Tricuspid
**CT**	Calcified Tricuspid
**RB**	Raphe Bicuspid
**NRB**	Non-Raphe Bicuspid
**AVIC**	Aortic Valve Interstitial Cell
***α*** **-SMA**	*α*-smooth muscle actin
**CytoD**	Cytochalasin-D
**N State**	Normal State
**I State**	Inactivated State

## Data Availability

The raw data supporting the conclusion of this article will be made available by the authors, without undue reservation.
