# Supplementary material for: Functional differences in human aortic valve interstitial cells from patients with varying calcific aortic valve disease
Source: Front Physiol. 2023 Jun 19;14:1168691. doi: 10.3389/fphys.2023.1168691 (PMC10316512; doi:10.3389/fphys.2023.1168691)
Supplement: Supplementary file 1 [file DataSheet1.pdf]

Supplementary Material

1 SUPPLEMENTARY DATA

1.1 Protrusion Tip Assignment

Regions of the AVIC surface mesh were apportioned into regions referred to as ”protrusion tips” and ”cell body”. The protrusion tips experienced the greatest magnitude of displacement and displaced in a ”piston-like” manner. The protrusion tips were isolating following the procedure discussed in 2.3.5, and were visually confirmed S1.

1.2 AVICs undergo deformation without change in volume or surface area

Using the point-to-point material correspondence, the volume and surface areas of each AVIC in both the Normal and CytoD-treated states were computed. There was no significant change in volume between states, groups, or sex (Fig. S3, S4). Thus, the spherical harmonic shape analysis is able to be performed with an assumption of isovolumetric deformation. In addition, the displacements at the protrusions are able to be understood as the depolymerization and deactivation of actin stress fibers, while the displacements of the cell body are considered compensatory to maintain cytoplasmic volume.

Although not essential for any assumptions for analysis, the surface area was also computed for each state and separated by group and sex. There was also no significant change in surface area from Normal to CytoD-treated states (Fig. S5, S6).

1.3 Porcine AVICs provide point of reference for magnitude of shape change

Healthy young adult porcine AVICs (pAVICs) from hearts sourced from a local abattoir were used to gauge the magnitude of shape change in diseased human AVICs. The diseased human AVICs had a much higher magnitude of shape change, in accordance with their degree of disease and presumed activation (Fig. S2).

2 SUPPLEMENTARY FIGURES

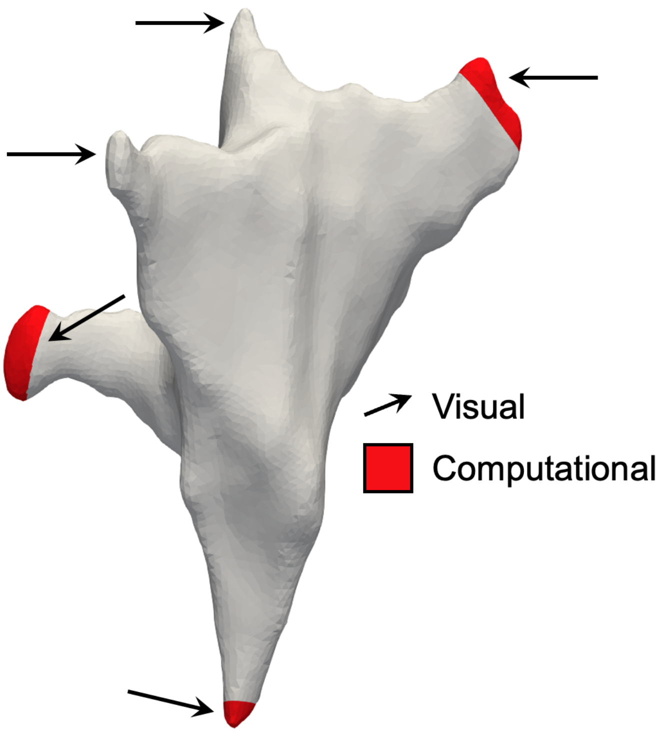

Figure S1. Identification of protrusion tips by visual and computational means. Computational methods capture the protrusions with the largest displacement for each cell.

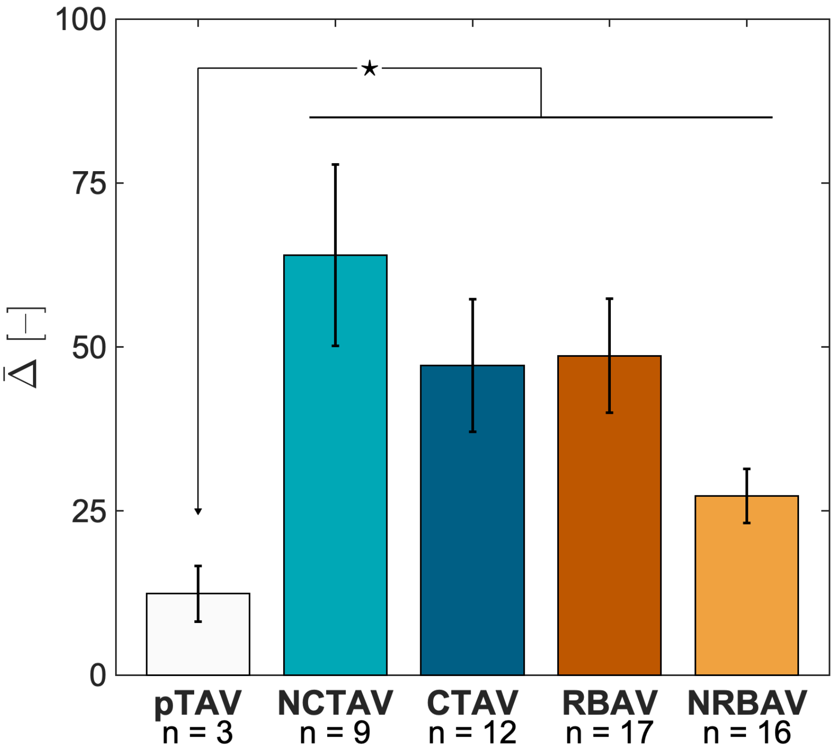

Figure S2. Total shape change from N to I. Porcine AVICs (pTAV) have the least amount of shape change in response to inactivation, showing a lower activation level than the diseased human AVICs. *P-value < 0.10

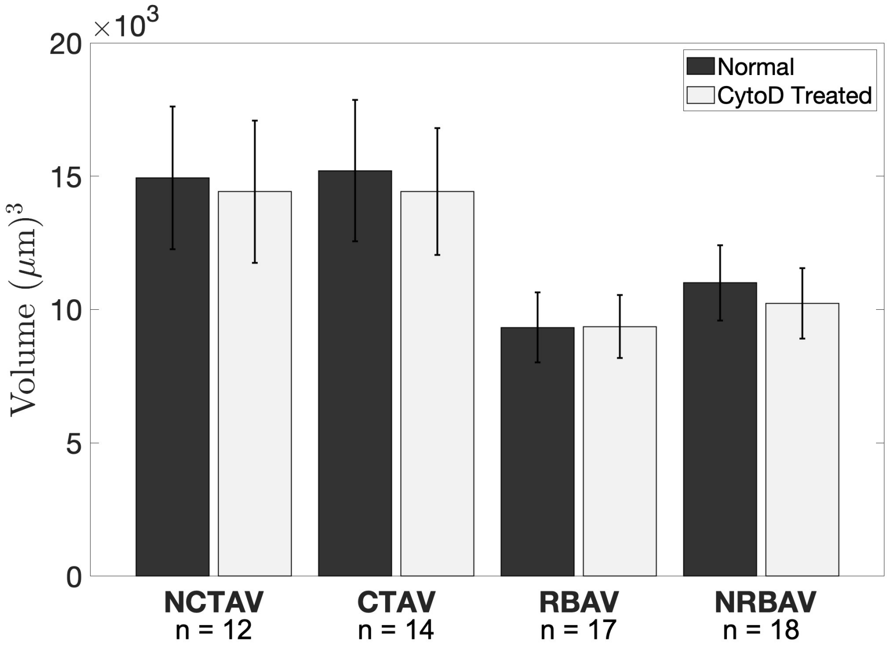

Figure S3. Change in AVIC volume from Normal to CytoD-treated separated by group. There is no significant change in volume for any group, nor are the AVIC volumes significantly different between groups.

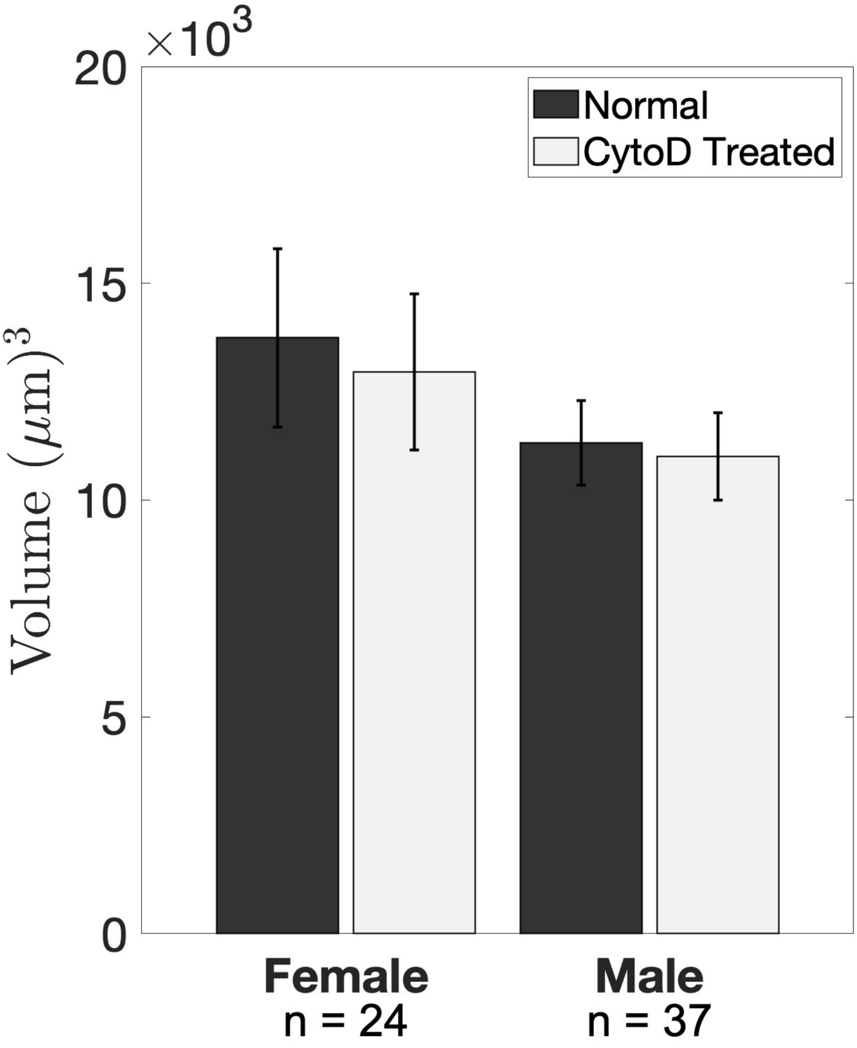

Figure S4. Change in AVIC volume from Normal to CytoD-treated separated by sex. There is no significant change in volume for either female or male, nor are the AVIC volumes significantly different between sexes.

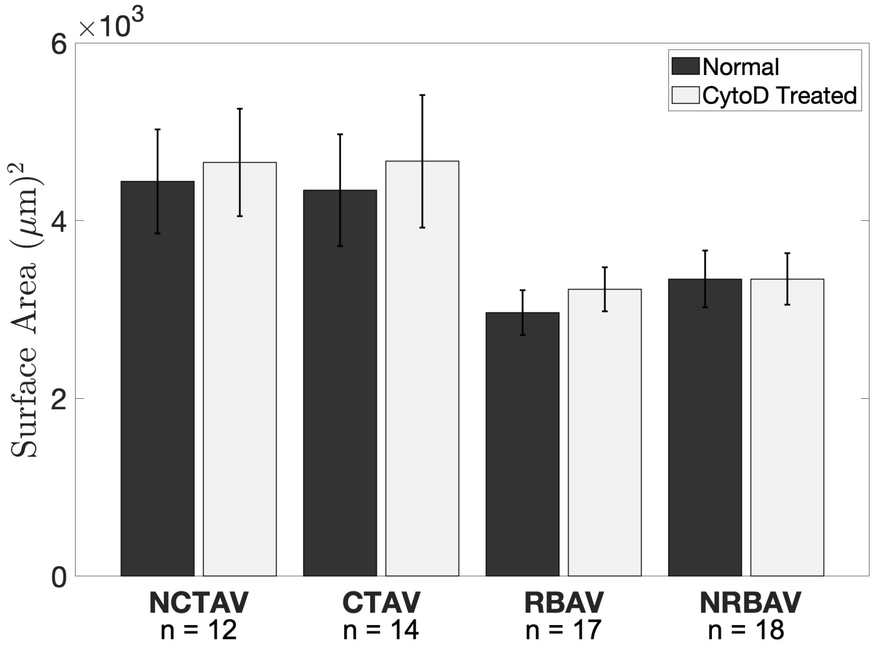

Figure S5. Change in AVIC surface area from Normal to CytoD-treated separated by group. There is no significant change in surface area for any group, nor are the AVIC surface areas significantly different between groups.

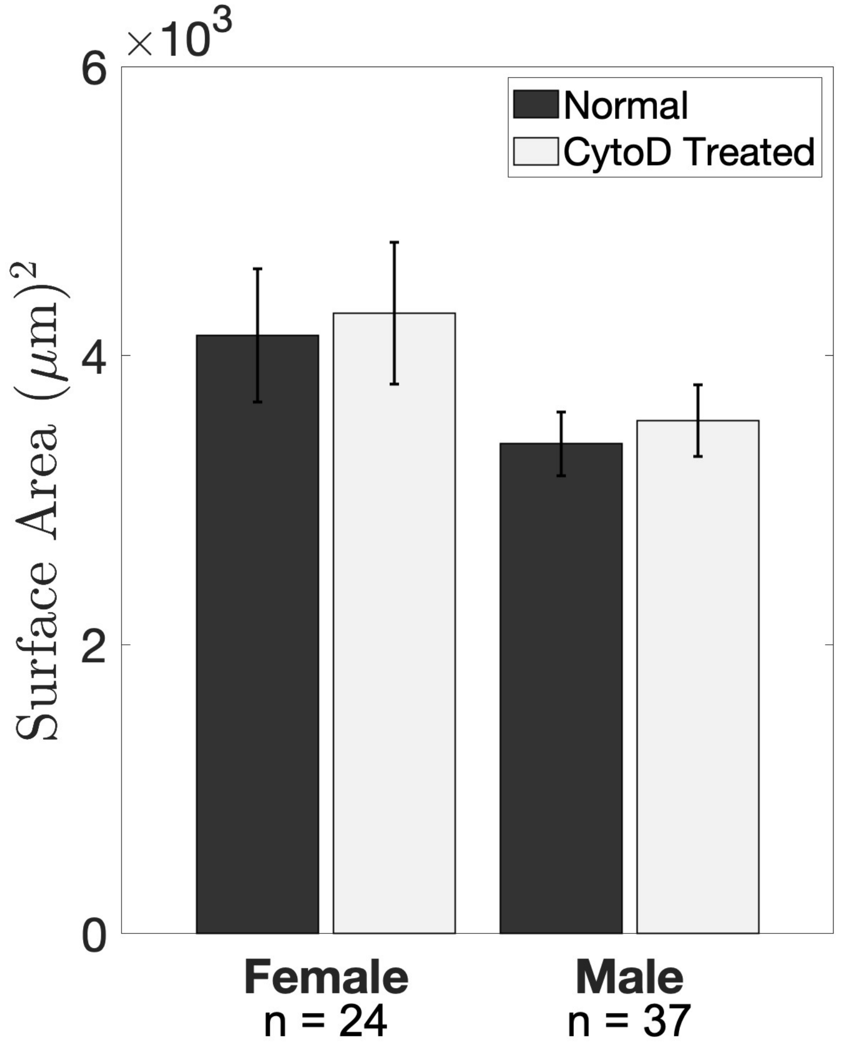

Figure S6. Change in AVIC surface area from Normal to CytoD-treated separated by sex. There is no significant change in surface area for either female or male, nor are the AVIC surface areas significantly different between sexes.
